# Identification of a new allele of the *Dw* gene causing brachytic dwarfing in peach

**DOI:** 10.1186/s13104-018-3490-7

**Published:** 2018-06-14

**Authors:** Celia M. Cantín, Pere Arús, Iban Eduardo

**Affiliations:** 1 IRTA, FruitCentre, Parc Científic i Tecnològic Agroalimentari de Lleida (PCiTAL), Edifici Fruitcentre, Parc de Gardeny, 25003 Lleida, Spain; 2 IRTA, Centre de Recerca en Agrigenòmica CSIC-IRTA-UAB-UB, Campus UAB, Cerdanyola del Vallès (Bellaterra), 08193 Barcelona, Spain

**Keywords:** Tree architecture, Molecular marker, *Prunus persica*, Marker-assisted selection

## Abstract

**Objective:**

Peach brachytic dwarfism determined by *Dwarf* gene (*Dw*) is an undesired trait segregating in some peach breeding programs. Recently, a single nucleotide polymorphism (SNP) mutation in the gibberellin insensitive dwarf 1 (GID1) peach gene causing brachytic dwarfism was described. In this research we wanted to validate this marker in an F_2_ population of the ‘Nectavantop’ peach cultivar (Nv) to include it as a marker assisted selection tool for peach breeding programs.

**Results:**

The observed segregation of the trait was in agreement with that of a recessive gene, the individuals homozygous for the recessive allele (*dwdw*) presenting the dwarf genotype. *Dw* was mapped to the distal part of linkage group 6 as previously described. The SNP marker based on the causal mutation previously described did not segregate in Nv F_2_ population. The sequence of the *GID1c* gene in Nv revealed a second SNP in its coding sequence which cosegregated with the dwarf phenotype. This SNP was predicted by the SNAP^2^ software to cause a major functional change and was validated in the dwarf peach cultivar ‘Small sunning’. These results suggest the existence of at least two independent mutations of the *Dw* gene causing the peach brachytic dwarf phenotype.

**Electronic supplementary material:**

The online version of this article (10.1186/s13104-018-3490-7) contains supplementary material, which is available to authorized users.

## Introduction

Until today different dwarf peach phenotypes have been described and different applications have been proposed for them, including the development of dwarf cultivars for intensive production, their use as ornamentals, and the use of genetically modified cultivars for the dwarfing genes to control fruit architecture [1]. The genetics of dwarf phenotypes has been studied and several major genes (*Dw*, *Dw2, Dw3)* have been identified [2–6]. Homozygous individuals for either *dw* or *dw2* display brachytic dwarfism (BD), presenting short internodes, thickened stems, reduced higher order branching, elongated leaves and normal fruit. Homozygous individuals for *dw3* are different from the previous ones and present narrow branches and willowy growth [7]. Dwarfing was also determined by a fourth gene (*N*) where the *nn* homozygote has short internodes, but *Nn* heterozygotes generate semi-dwarf individuals [8, 9]. Another dwarfing phenotype, recently described by Lu et al. [10], is the temperature-sensitive semi-dwarf (*Tssd*) locus that regulates internode length. The presence of the dominant *Tssd* allele determines short internode length at temperatures below 30 °C. *Dw* and *Tssd* have been mapped to the distal part of chromosome 6 [6, 11] and to the proximal part of chromosome 3 [10], respectively. Only *Dw* has been cloned [11], being the *dw* allele generated by a non-sense mutation resulting in a non-functional product of the gibberellic acid (GA) receptor *PpeGID1c* gene [11]. *PpeGID1c* is annotated as *Prupe.6G332800* in the peach genome v2.0 and as *ppa018174* in v1.0. For the other dwarfing genes there is no information on their map positions or markers tightly linked to them.

BD individuals are often found to be segregating in peach breeding programs and they are usually discarded. Molecular markers such as the gid1c SNP described by Hollender et al. [11], can be used as tools for early identification and removal of dwarf plants in peach breeding programs or to avoid crosses between carriers of the *dw* allele. Several molecular markers for other traits such as fruit shape [12], fruit acidity [13] and the slow ripening phenotype [14], among others [15], are already being used routinely for marker-assisted selection (MAS) in peach [16]. In this work, we describe the identification of a new allele from the *Dw* gene producing dwarf individuals found in ‘Nectavantop’ and ‘Small sunning’ cultivars.

## Main text

### Methods

#### Plant material and DNA extraction

A dwarf segregating population of 77 individuals obtained from the open pollination (OP) of ‘Nectavantop’ (Nv) cultivar was used for this study. Trees were planted on their own roots in 2013 in the plots at the IRTA Experimental Station at Gimenells (Lleida, Spain). Trees were visually classified as normal or dwarf during 2 consecutive years (2014 and 2015), that corresponded to the first and second year after planting. Concurrently, one tree of dwarf cultivars ‘Bonanza’ (Bo) and ‘Small sunning’ (Ss) were grown in 50 L pots in the IRTA greenhouse at Torre Marimon (Caldes de Montbui, Spain). DNA from the 77 genotypes of Nv⊗ and Nv, Bo and Ss was extracted from young leaf tissue using the Doyle and Doyle [17] protocol adapted to 96 well plates.

#### Genotyping

For simple sequence repeat (SSR) genotyping, polymerase chain reactions (PCR) reactions were carried out in a PE9700 Thermal Cycler (PE/Applied Biosystems, Foster City, California, USA) in a volume of 10 μl, containing 20 ng of peach genomic DNA, 1× NH4-based Reaction Buffer, 1.5 mM MgCl2, 0.5 mM dNTPs, 0.25 μM of each primer and 1 U of BIOTAQ (Bioline). Forward primers were labelled with a fluorochrome (FAM, VIC, NED or PET). PCR amplifications were carried under the following conditions: 1 min at 95 °C, 30 cycles of 15 s at 95 °C, 15 s at the appropriate annealing temperature, and 30 s at 72 °C, followed by a 5 min extension at 72 °C. Products were analyzed by capillary electrophoresis using the ABI/Prism 3130xl (PE/Applied Biosystems) sequencer as in Aranzana et al. [18]. CPP SSRs were genotyped using the multiplex-ready PCR as described in Donoso et al. [19]. A SNP located within the candidate gene *GID1c* and called gid1c was described by Hollender et al. [11] as the causal mutation from the BD phenotype. Primers to amplify a DNA fragment containing gid1c were designed (Table 1) to genotype it by Sanger sequencing. PCR conditions for fragment amplification were the same than those of the SSRs, but using 1 min of extension. PCR fragments were visualized in ethidium bromide agarose gels under UV light for amplification pattern verification, later on they were sequenced in the capillary sequencing service from CRAG (http://www.cragenomica.es/core-facilities/capillary-sequencing) using standard protocols.

**Table 1:** Primers of the five new SSRs used to map the dwarf trait and the two primers for SNP genotyping

Primer name	Primer sequence	Genomic position
CPP24554-F	TGAGGGAAGTTTGGTTGCTC	6:28182155
CPP24554-R	AATTGAGATGAATGGGGCGC	6:28182388
CPP24650-F	GGCACGTGAGAGGGATATGA	6:28892277
CPP24650-R	AACTTAAGTCAGCGGCAGGAT	6:28892516
CPP24654-F	GGGGTAAATAAGACTTTTGACAACT	6:28897444
CPP24654-R	AGTCGGTCTAAGGTGTGAAAACA	6:28897685
CPP24664-F	ATTAAACCACAGACGCACGG	6:28996880
CPP24664-R	TGACCATGTGCGTATCATTTGT	6:28997069
CPP24681-F	TTCCCTGCTTGACACGTGTA	6:29123267
CPP24681-R	ACACTCACTCTGTCTTCCGGT	6:29123396
ppa018174-F	AACTGGCCTGCTTACTCGAA	6:28967552
ppa018174-R	GCCAGTCCTGAACAAGATCC	6:28966891

Genomic positions are according to the *Prunus* genome v2.0

#### Linkage mapping

Nv⊗ was first screened with four SSR markers (UDP98-412, MA014a, CPPCT030, CPPCT021) known to be located at the distal end of linkage group 6 (G6) where the peach *Dw* gene had been previously mapped [6, 11]. Five additional SSRs were developed at this region using the peach genome sequence v2.0 (http://www.rosaceae.org), and the set of SSRs described in http://services.appliedgenomics.org/projects/drupomics/gbrowse/. The new SSRs were noted as CPP followed by the number assigned to this SSR in the IGA (Istituto di Genomica Applicata) annotation of the peach genome v1.0 (Table 1). Six out of the nine SSRs screened in Nv were heterozygous and were genotyped in the progeny. The other three SSRs (MA014a, CPPCT021 and CPP24554) were homozygous in Nv and could not be used for mapping. Eight individuals were found to contain alleles different from those of Nv in at least one marker indicating that they came from cross pollination and were discarded from the dataset. The remaining 69 individuals were considered as true F_2_ individuals and used for map construction.

A linkage map was constructed using JoinMap v.4.1 [20] software. Groups were established with a LOD ≥ 3.0 and the map was calculated with the Kosambi distance function. Linkage group nomenclature follows the *Prunus* reference map (T × E) [21]. MapChart 2.1 software [22] was used to draw the map. To predict the effects of a new sequence variant identified in this research on the function of the *PpeGID1c* gene product we used the SNAP^2^ software program [23].

### Results

#### Genetic mapping of Dw

**Fig. 1:**
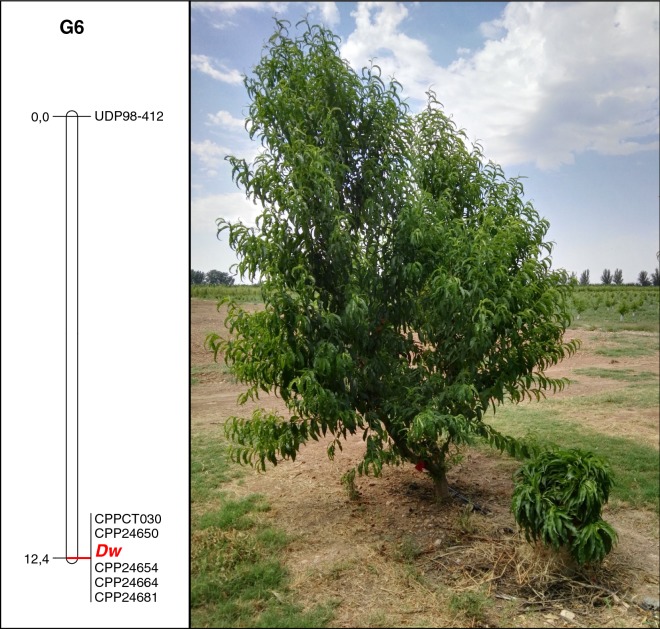
Linkage map of the Nv⊗ population showing the position of the *Dw* gene. Genetic distances are expressed in cM. On the right, two Nv⊗ individuals on the third year after planting, where the difference between the brachytic dwarf phenotype and the normal phenotype can be observed

Dwarf trees from Nv population were much smaller and with shorter internodes than normal trees (Fig. 1). The description of the dwarf trees corresponded very well with that of Hollender et al. [11], and differences between normal and dwarf trees were already evident from the beginning of seedling development. Twenty-two out of the 69 F_2_ individuals were dwarf, representing a 47:22 segregation, which fits with a 3:1 segregation (χ^2^ = 1.74; n.s.). This suggests that the BD trait was controlled by a single gene (*Dw*) with *dw* as a recessive allele, as previously described [6, 11]. *Dw* was codified according to that and was included with the other six SSRs in the mapping dataset. A single linkage group (LG), corresponding to Prunus LG6, was identified (Fig. 1), where *Dw* was cosegregating with markers CPPCT030, CPP24650, CPP24654, CPP24664 and CPP24681, spanning a genomic region of approximately 400 Mb according to the *Prunus* genome v2.0, and UDP98-412 was 12.4 cM apart.

#### SNP identification in the GID1c candidate gene

**Fig. 2:**
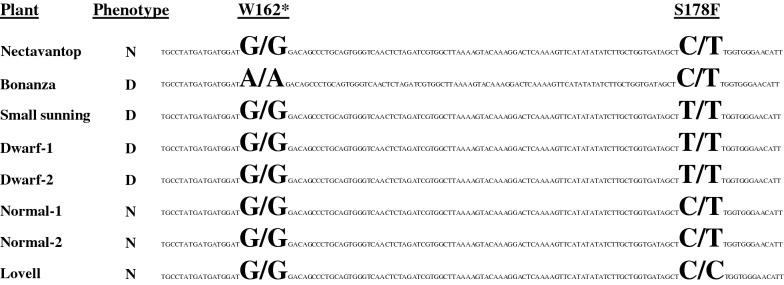
Sequence fragment from *GID1c* (*Prupe.6G332800*) gene containing both SNPs, w162* and the new S178F SNP described in this work. Genotypes of the different cultivars for both SNPs are shown. Phenotypes are indicated as normal (N) or dwarf (D)

The chromosomal region delimited by the markers cosegregating with *Dw,* co-locates with that of the candidate gene *GID1c* reported by Hollender et al. [11]. These authors demonstrated that the *A/A* genotype from SNP-W162* was responsible for the BD trait [11]. This SNP is physically located between markers CPP24654 and CPP24664, suggesting that it could be also responsible for the phenotypic variability observed in Nv F_2_ population. To validate that option, a DNA fragment of 662 bp around the SNP-W162* was amplified and sequenced in ‘Nectavantop’, ‘Bonanza’, ‘Small sunning’ and two normal and two dwarf Nv F_2_ individuals. These sequences contained two SNPs, one corresponding to the SNP-W162* and another one 87 bp further, which we named SNP-S178F. The only individual presenting the dwarf allele (*A/A*) in the SNP-W162*, was ‘Bonanza’. The new SNP, SNP-S178F, was found to have the *T/T* genotype in the two dwarf individuals from the Nv F_2_ population and in ‘Small sunning’, was heterozygous (*C/T*) in ‘Nectavantop’ and the normal size F_2_ individuals, and was homozygous *C/C* in ‘Bonanza’ (Fig. 2).

#### SNP effect prediction

The new SNP-S178F results in an amino acid substitution of a serine (TCT) for a phenylalanine (TTT) in the gibberellic acid receptor protein encoded by the *GID1c* gene. To predict the possible effect of this mutation in the product of this gene, its sequence was analyzed with SNAP^2^ software. The SNP-S178F is located in a region of 12 amino acids where mutations can have a high effect on the protein functionality. In our case, the substitution of a Serine by a Phenylalanine presents a high score only exceeded by three other amino acidic changes (Additional file 1), suggesting that the effect of this mutation is sufficiently important to disrupt the functionality of the transcribed protein with phenotypic effects that are indistinguishable from the allele detected by the SNP-W162*, originally found in the Japanese cultivar ‘Juseito’, which produces a stop codon [11].

### Discussion

Our results indicate that the BD phenotype segregating in the Nv F_2_ population, although mapping in the same position at the distal part of G6, is not caused by the SNP described by Hollender et al. [11], but by a different allele. As it occurs for other peach genes that have been characterized at the molecular level in peach, such as the white vs. yellow flesh color [24], various alleles of different origin may cause similar recessive phenotypes. One of the consequences of this is that selection for markers based on the causal allele may be useful for only one transect of the variability of the species and are useless for the other. Knowledge of the pedigree of the individuals sampled is then a requirement for the adequate choice of markers. This shows also that even in a species with a low level of variability like peach [25], multiallelic series may be common for genes with phenotypic effects. While in the case of the yellow vs. white gene may be attributed to the amplification of variability caused by human selection for characters related with the edible part of the plant in crop species, it appears not to be the case for the dwarf gene, where the homozygous recessive dwarf individuals are usually rejected.

## Limitations

The main limitations of our results are the low resolution of our mapping population, probably because of the reduced number of individuals used and the low number of cultivars where both SNPs markers, W162* and S178F, could be validated.

## Additional file

**Additional file 1.** Functional effects in the protein encoded by the *GID1c* (*Prupe.6G332800*) sequence predicted by the SNAP^2^ software. (A) Global view of effects in the full protein sequence. The Serine affected by the gid1c2 SNP is marked with an arrow. (B) Scores of the different possible mutations in the Serine affected by the gid1c2 SNP. The specific change of a Serine for a Phenylalanine is marked with an arrow.

## Abbreviations

BD: brachytic dwarfism
Bo: Bonanza
DNA: deoxyribonucleic acid
Dw: dwarf
GA: gibberellic acid
GID1: gibberellin insensitive dwarf 1
LG: linkage group
MAS: marker assisted introgression
Nv: nectavantop
PCR: polymerase chain reaction
SNP: single nucleotide polymorphism
Ss: Small sunning
SSR: simple sequence repeat
Tssd: temperature-sensitive semi-dwarf
