# Supplementary material for: Identification of a new allele of the Dw gene causing brachytic dwarfing in peach
Source: BMC Res Notes. 2018 Jun 14;11:386. doi: 10.1186/s13104-018-3490-7 (PMC6000960; doi:10.1186/s13104-018-3490-7)
Supplement: Supplementary file 1 — Additional file 1. Functional effects in the protein encoded by the GID1c (Prupe.6G332800) sequence predicted by the SNAP2 software. (A) Global view of effects in the full protein sequence. The Serine affected by the gid1c2 SNP is marked with an arrow. (B) Scores of the different possible mutations in the Serine affected by the gid1c2 SNP. The specific change of a Serine for a Phenylalanine is marked with an arrow. [file 13104_2018_3490_MOESM1_ESM.pdf]

A)

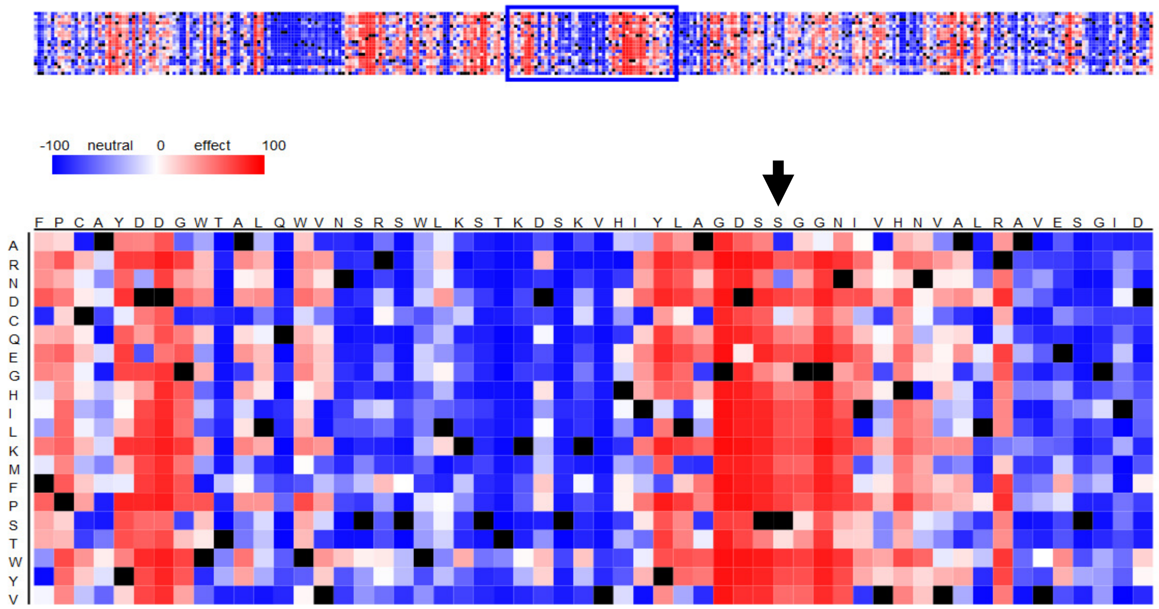

B)

Wildtype Amino Acid	Position	Variant Amino Acid	Predicted Effect	Score	Expected Accuracy
S	178	A	neutral	-85	93%
S	178	R	effect	63	80%
S	178	N	neutral	-52	78%
S	178	D	effect	74	85%
S	178	C	neutral	-11	57%
S	178	Q	effect	51	75%
S	178	E	effect	71	85%
S	178	G	effect	31	66%
S	178	H	effect	53	75%
S	178	I	effect	70	85%
S	178	L	effect	69	80%
S	178	K	effect	72	85%
S	178	M	effect	62	80%
S	178	F	effect	73	85%
S	178	P	effect	75	85%
S	178	S	neutral	-99	97%
S	178	T	effect	13	59%
S	178	W	effect	80	91%
S	178	Y	effect	70	85%
S	178	V	effect	58	75%
